# Selective Spectrum Antibiotic Modulation of the Gut Microbiome in Obesity and Diabetes Rodent Models

**DOI:** 10.1371/journal.pone.0145499

**Published:** 2015-12-28

**Authors:** Deepak K. Rajpal, Jean-Louis Klein, David Mayhew, Joyce Boucheron, Aaron T. Spivak, Vinod Kumar, Karen Ingraham, Mark Paulik, Lihong Chen, Stephanie Van Horn, Elizabeth Thomas, Ganesh Sathe, George P. Livi, David J. Holmes, James R. Brown

**Affiliations:** 1 Computational Biology, Target Sciences, Research and Development, GlaxoSmithKline, Collegeville, Pennsylvania, United States of America; 2 Target and Pathway Validation, Target Sciences, Research and Development, GlaxoSmithKline, Collegeville, Pennsylvania, United States of America; 3 Enteroendocrine Discovery Performance Unit, Research and Development, GlaxoSmithKline, Research Triangle Park, North Carolina, United States of America; 4 Antibacterial Discovery Performance Unit, Research and Development, GlaxoSmithKline, Collegeville, Pennsylvania, United States of America; National Institute of Agronomic Research, FRANCE

## Abstract

The gastrointestinal tract microbiome has been suggested as a potential therapeutic target for metabolic diseases such as obesity and Type 2 diabetes mellitus (T2DM). However, the relationship between changes in microbial communities and metabolic disease-phenotypes are still poorly understood. In this study, we used antibiotics with markedly different antibacterial spectra to modulate the gut microbiome in a diet-induced obesity mouse model and then measured relevant biochemical, hormonal and phenotypic biomarkers of obesity and T2DM. Mice fed a high-fat diet were treated with either ceftazidime (a primarily anti-Gram negative bacteria antibiotic) or vancomycin (mainly anti-Gram positive bacteria activity) in an escalating three-dose regimen. We also dosed animals with a well-known prebiotic weight-loss supplement, 10% oligofructose saccharide (10% OFS). Vancomycin treated mice showed little weight change and no improvement in glycemic control while ceftazidime and 10% OFS treatments induced significant weight loss. However, only ceftazidime showed significant, dose dependent improvement in key metabolic variables including glucose, insulin, protein tyrosine tyrosine (PYY) and glucagon-like peptide-1 (GLP-1). Subsequently, we confirmed the positive hyperglycemic control effects of ceftazidime in the Zucker diabetic fatty (ZDF) rat model. Metagenomic DNA sequencing of bacterial 16S rRNA gene regions V1-V3 showed that the microbiomes of ceftazidime dosed mice and rats were enriched for the phylum Firmicutes while 10% OFS treated mice had a greater abundance of Bacteroidetes. We show that specific changes in microbial community composition are associated with obesity and glycemic control phenotypes. More broadly, our study suggests that *in vivo* modulation of the microbiome warrants further investigation as a potential therapeutic strategy for metabolic diseases.

## Introduction

Incidents of metabolic diseases, in particular obesity and type 2 diabetes mellitus (T2DM), are rising to the level of global epidemics [1]. With limited available treatment options, new therapeutic strategies are necessary for the control of these diseases. The roles of gastrointestinal tract (GIT) microbiota in metabolic and inflammatory diseases are intensive areas of recent investigation as an alternative therapeutic modality [2,3]. Both animal and clinical human studies suggest that energy conversion as well as pro-inflammatory effects of the GIT microbiome (the genome collective of GIT microbiota) have a role in the progression and severity of obesity and diabetes. For example, the transmissibility of total body fat phenotype by so-called ‘obese microbiota’ over ‘lean microbiota’ in germ-free mice, clearly point to GIT microbes as key contributors to the pathophysiology of obesity [4]. Supporting this view are studies that show GIT microbial communities of healthy individuals significantly differ from those of obese [5,6] or diabetic [7,8] subjects.

The causal relationship between the composition of GIT microbiome and the obesity/diabetes human phenotype is still an open question. Some studies suggest that changes in the relative abundances of bacterial phyla, specifically low Bacteroidetes and high Firmicutes abundances, are associated with increased weight-gain and obesity [9] while other reports suggest the opposite ratio, high Bacteroidetes to low Firmicutes, has this effect [10]. Elevated levels of Actinobacteria [11] or overall lower bacterial community richness [6] have also been reported to be linked to increased severity of obesity. Other studies point to specific bacterial species such as the Verrucomicrobia, *Akkermansia muciniphila*, as potential indicators of a healthy human microbiome due to their role in maintaining the protective mucus lining of the intestines [12,13] and facilitating potential immune-modulatory cross-talk with human gut luminal receptors [14].

Probiotics, prebiotics and antibiotics have used in both animal studies and human clinical trials to assess the effects of changing the GIT microbiome on obesity and diabetes disease phenotypes. Dosing with antibiotics norfloxacin and ampicillin enhanced glucose tolerance in genetic and diet-induced obesity (DIO) mouse models as well as insulin resistant mice [15]. The prebiotic oligofructose saccharide (OFS), a short-chain form of inulin plant fiber, is widely used as a supplement in food products and infant formula with demonstrated modest weight loss effects in adults [3]. In humans, OFS increases short-chain fatty acids, L-cells and gut peptides such as peptide tyrosine tyrosine (PYY) and reduces ghrelin [16]. The immune-modulatory effects of OFS are believed to be driven through the gut microbiota by increasing levels of Bifidobacteria and Lactobacillus [17].

A major caveat of previous microbiome studies involving prebiotic or antibiotic administration is the non-specificity of these agents with respect to bacterial species spectrum. Arguably, using narrow or focused bacterial spectrum agents to selectively “knock-out” or “knock-down” microbiota species in model systems could help to decipher microbiome-disease causality. The goal of our study was to better determine the relationship between specific GIT microbial communities and the obesity/diabetes phenotype. To accomplish this, we attempted the *in vivo* modulation of the microbiome in the DIO mouse model using two antibiotics with markedly different bacterial spectra. The antibiotic vancomycin specifically inhibits cell wall synthesis in Gram-positive bacteria with little effect on Gram-negative bacteria due to differences in their outer membrane structures [18]. In contrast, ceftazidime is a third generation cephalosporin which inhibits cell wall synthesis in Gram-negative bacteria [19]. We also compared the effects of these antibiotics with that of the prebiotic OFS (10% supplement) and measured changes in mouse metabolic phenotypes, hormones, biochemistry and GIT microbiome 16S ribosomal RNA (rRNA) gene profiles. Furthermore, we confirmed our findings in the Zucker diabetic fatty (ZDF) rat, a leptin gene deficient (*lep-/lep-*) animal model widely used for diabetes studies [20].

Our collective results show that ceftazidime dosing leads to enhanced weight loss and hyperglycemic control potentially through an increase in the relative proportion of Firmicutes (Gram-positive bacteria) to Proteobacteria (Gram-negative bacteria). In contrast, mice treated with the prebiotic OFS showed weight loss and increased Bacteroidetes relative abundances but no significant changes in glucose metabolism. Our study illustrates the utility of chemogenomics approaches to better understand the relationships of between GIT microbial ecosystems and metabolic disease phenotypes.

## Materials and Methods

### Animal handling

All procedures were performed in compliance with the Animal Welfare Act, USDA regulations and approved by the GlaxoSmithKline Institutional Animal Care and Use Committee. Male diet-induced obese (DIO) C57BL/6 mice and age-matched lean controls, approximately 14 weeks of age, were purchased from Taconic (Germantown, NY). The DIO mice were group-housed and fed a high fat diet (60% fat by kcal) (D12492, Research Diets, New Brunswick, NJ) by the vendor from the time of weaning. Upon receipt, all mice were single housed under controlled conditions (12:12 light-dark cycle, 24°C and 50% relative humidity) with free access to rodent chow. DIO mice were fed a 45% high fat pelleted diet (D12451, Research Diets, New Brunswick, NJ), and lean mice were fed Lab diet 5001, (PMI Nutrition International, Brentwood, MO). After 2 weeks acclimation the mice were weighed, fat and non-fat mass body composition measurements were made using quantitative magnetic resonance (QMR) (Echo Medical Systems, Houston, TX) and the mice were randomized into treatment groups (n = 10) with similar mean body weight and body composition.

Male Zucker diabetic fatty rats (ZDF-*Lepr* ^*fa*^/Crl) were purchased from Charles River (Raleigh, NC) and housed under controlled conditions (12:12 light-dark cycle, 24°C and 50% relative humidity) with free access to rodent food (Purina 5008, Harlan Teklad, Indianapolis, IN). All rats arrived at seven weeks of age (± 3 days). All animals were euthanized under isoflurane anesthesia via cervical dislocation (mouse) or cutting the heart open (rat).

### DIO mouse study design

DIO mice were acclimated to dosing, with a three day saline vehicle lead-in period, and to D12451 meal chow, for two days, prior to drug treatment. DIO mice were fed chow with either no supplement (vehicle) or chow supplemented with 10% OFS, vancomycin (VAN) or ceftazidime (CEF) (each antibiotic at three dose levels 50 mg/kg, 150 mg/kg or 500 mg/kg) daily at 4 pm over a period of 2 weeks. Formulations of VAN (Akorn-Strides, LLC, Buffalo Grove, IL) and CEF (Sagent Pharmaceuticals, Inc., Schaumburg, Illinois) and OFS (Beneo-Orafti Inc, Morris Plains, NJ) mixed into Research Diets D12451 meal chow were prepared weekly. Body weight was measured three times a week and food intake every weekday. After 2 weeks body compositions measurements were made and whole blood samples were collected by cardiac puncture under isoflurane anesthesia from non-fasting animals. Serum levels of glucose (GLU), total cholesterol (CHOL), HDL-cholesterol (HDL), triglycerides (TRIG), non-esterified fatty acids (NEFA), beta hydroxybutyrate (b-HBA), glycerol (GLY), aspartate aminotransferase (AST), alanine aminotransferase (ALT), total bilirubin (TBIL), blood urea nitrogen (BUN), creatinine (CREA), creatine kinase (CK), total protein (TP), albumin (ALB), amylase (AMY), and lipase (LIP) as well as plasma levels of insulin, amylin, leptin, ghrelin, PP, PYY, tGLP-1 and aGLP-1were determined. Heart, liver, pancreas, kidney, GIT tract (stomach, duodenum, jejunum, ileum, colon, ceacum), WAT (epididymal), BAT (hibernating gland) were collected for macroscopic and microscopic evaluation and ceca contents were collected, snap frozen in liquid nitrogen and stored at– 80°C for microbiome analysis.

### ZDF rat study design

After a one-week acclimation period, rats were anesthetized with isoflurane (Abbott Laboratories, IL) and tail-vein blood samples were collected at 9 AM without fasting. Blood glucose levels were measured using a glucometer (Bayer, Leverkusen, Germany). In order to ensure balanced treatment groups, ZDF rats were assigned to two treatment groups based upon baseline glucose: vehicle (0.5% HPMC, 0.1% Tween80) and CEF (500mg/kg). All treatments were given via oral gavage twice a day and animals were followed for two weeks with blood samples collected from tail vein at the end of each week at 9:00 AM without fasting. Fecal samples were collected for 24 hours on day 12.

### Biochemical analysis

Plasma and serum were prepared using EDTA and T-MG tubes, respectively [Terumo Medical Corporation, Elkton, MD]. EDTA tubes used to prepare plasma for hormone analysis contained 50 μM DPP IV inhibitor [Millipore, St. Charles, MO] and a protease inhibitor cocktail [Sigma-Aldrich, St. Louis, MO]. Clinical chemistry analysis was performed by standard techniques using an Olympus AU640 Clinical Chemistry analyzer (Olympus America Inc., Melville, NY). Insulin, amylin, leptin, ghrelin, GIP and PYY were analyzed using Milliplex MAP Mouse Gut Hormone Panel and MSD assays were used for measurement of aGLP-1 and tGLP-1 (Meso Scale Discovery, Gaithersburg, MD). Glycated hemoglobin (HbA1c) in blood hemolysates was analyzed with a Primus ultra2 liquid affinity chromatograph (Trinity Biotech, Jamestown, NY).

### Fecal extraction

Fecal samples were air-dried for five days and extracted in Methanol-KOH (300 mM) at 60°C for 24 hours. Fecal extract was then mixed with 150mM Mg2SO4 (1:1). After centrifugation, the supernatant was saved and submitted for analysis.

### Statistical analysis

Data were analyzed by one-way ANOVA with post-hoc Dunnett’s method (DIO mouse study) or Student’s t-test (ZDF rat study) to compare all treatments to vehicle control. A P-value ≤ 0.05 was considered significant (JMP statistical software, SAS Institute, Cary, NC).

### Microbiome analysis

Bacterial DNA extraction from fecal material, DNA sequencing of 16S rRNA V1-V3 region, reagent controls and microbiome data analysis were as previously described [21]. Raw sequence data were processed and analyzed using the QIIME software package [22]. Reads shorter than 200 base pairs, longer than 1,000 base pairs, with more than 6 homopolymers, or with average quality less than 25 were discarded. Reverse primers were truncated from sequences and a maximum of one mismatch was allowed when matching the reverse primer. Chimeric sequences were identified and removed from the dataset using ChimeraSlayer [23]. The “closed-reference” QIIME protocol was used with the UCLUST method [24] to select operational taxonomic units (OTUs). Sequences with at least 97% identity were clustered together. A representative sequence from each cluster was used to identify bacterial taxa from the May 2013 edition of the Greengenes 16S rRNA database [25]. OTUs containing fewer than 2 sequences were discarded, and sequences with less than 60% similarity to the Greengenes database were also discarded to remove potential contaminants from the dataset. Potential changes in the microbiome at the functional level were determined using the software PICRUSt [26] with default settings, the KEGG (Kyoto Encyclopedia of Genes and Genomes) database release 70.0 [27,28]. Human specific pathways were removed from the results to focus on true bacterial pathways. False discovery rate (FDR) corrected P-values < 0.05 were used to determine statistical significance for all analysis [29]. R [30] and custom scripts were used for statistical analysis. Study sequence data are deposited in the National Center for Biotechnology Information Sequence Read Archive under accession number SRP059837.

## Results

### Antibiotic and prebiotic effects in DIO mice

Our study consisted of nine treatment cohorts each with ten mice (n = 10); one cohort was on lean diet (lean) while the others were on a diet-induced obesity (DIO) regimen. The DIO mice received either chow with no supplement (vehicle) or chow supplemented with 10% OFS, VAN or CEF with the latter two antibiotics dosed in three different regimens of 50, 150 and 500 mg/kg. VAN and CEF were selected as the most specific commercially available bacteriocidal antibiotics against Gram-positive and Gram-negative bacteria, respectively, based on published reports [18,19] and our unpublished minimum inhibitory concentration (MIC) screening data. Mouse body weight changes and food intake were regularly monitored. At the end of 14 days, mice were sacrificed for further organ specific pathohistology, biochemistry and microbiome analyses.

**Fig 1:**
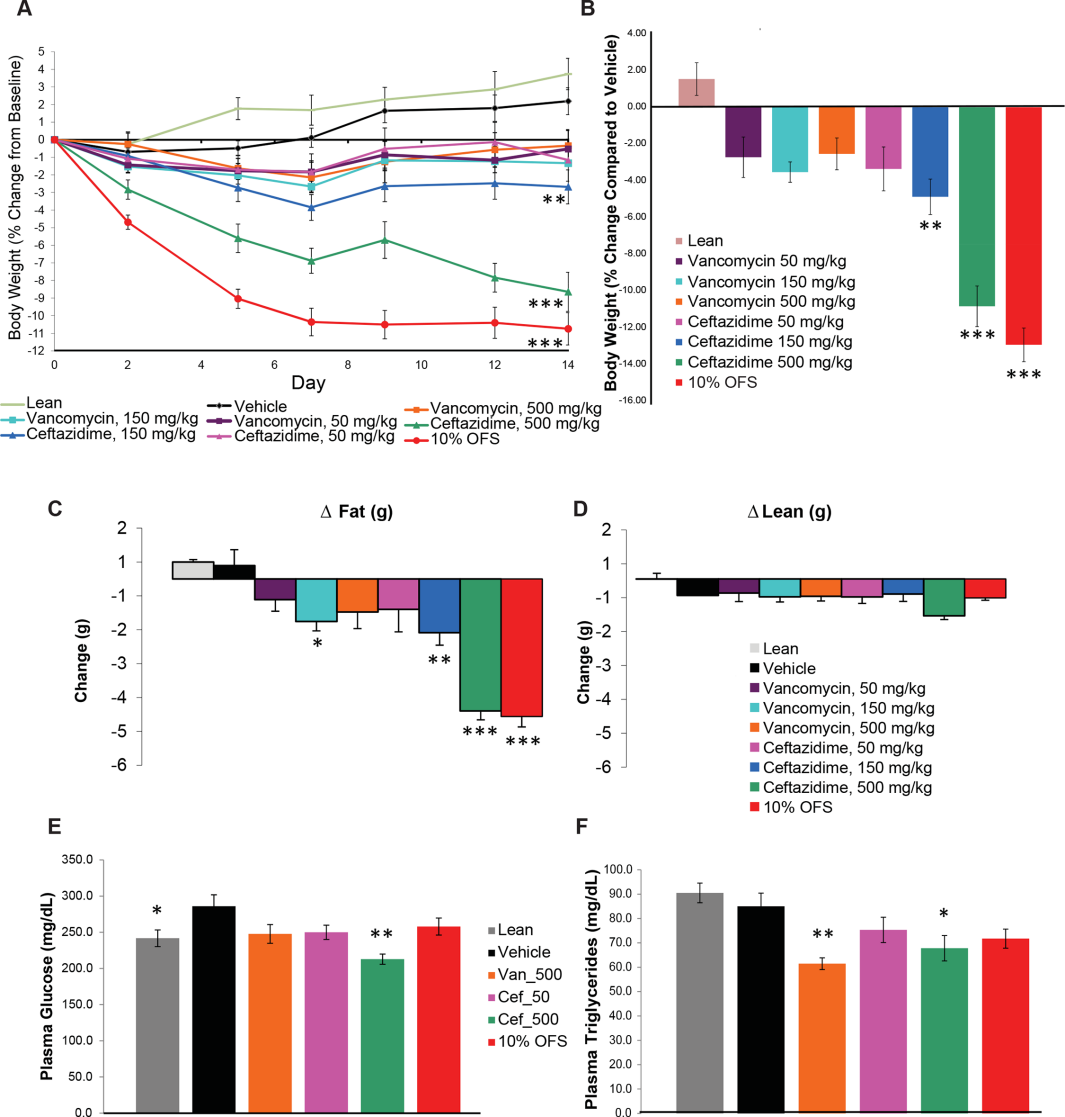
Changes in body weight, glucose and triglycerides for mice given 10% oligofructosaccharide (OFS) supplement or one of three different dosing regimens of the antibiotics vancomycin and ceftazidime. (A) Percent change in body weight from baseline over 14 days. (B) Overall body weight change compared to vehicle at 14 days. Relative to baseline the changes after 14 days in (C) overall body fat and, (D) lean muscle mass. (E) Plasma glucose and, (F) triglycerides at 14 days as measured for treatment cohorts of lean, vehicle, vancomycin 500 mg/kg (VAN_500), ceftazidime 50 (CEF_50) and 500 mg/kg (CEF_500) and 10% OFS. * P < 0.05, ** P < 0.01, *** P < 0.001 obtained from ANOVA, Dunnett’s square. All error bars denote s.e.m.

Significant weight changes relative to baseline (Fig 1A) and the vehicle (Fig 1B) were observed at the end of study in descending order for three treatments, 10% OFS (P < 0.0001, Dunnett’s ANOVA), CEF 500 mg/kg (P < 0.0001) and CEF 150 mg/kg (P = 0.004). No significant differences in weight loss were observed for any of the three VAN dose regimens or CEF 50 mg/kg although there was a trend of increasing weight loss corresponding with increasing CEF dosage. Food intake initially declined for 10% OFS and CEF treated mice for Days 2–5 but attained similar levels across all treated animals by Day 14 (S1A Fig). Overall food intake was significantly lower for 10% OFS (P < 0.0001, Dunnett’s ANOVA), CEF 500mg/kg (P < 0.0001), CEF 150mg/kg (P = 0.002) and CEF 50 mg/kg (P = 0.011) cohorts (S1B Fig). Reduction in body fat (g) relative to baseline was significantly higher for mice treated with 10% OFS (P < 0.0001, Dunnett’s ANOVA), CEF 150mg/kg (P = 0.005), CEF 500mg/kg (P < 0.0001) and VAN 150 mg/kg (P = 0.05) but not for other doses of VAN or CEF (Fig 1C and 1D). Percent body fat at day 14 was lower for lean mice (P < 0.0001, Dunnett’s ANOVA), 10% OFS (P < 0.0001) and CEF 500 mg/kg (P < 0.0001) treated animals versus vehicle (S1C Fig).

All animals were healthy at the end of study and pathological examination did not reveal any toxicity of target organs which included heart, liver, pancreas, kidney, gastrointestinal tract, epididymal and brown adipose tissue. Well-formed stools were produced by all animals throughout the study although mice treated the two highest doses of VAN and CEF (150 mg/kg and 500 mg/kg)) had formed feces that were red-brown in color. Briefly, decreased cytoplasmic lipid droplets/content was observed in the liver and brown adipose tissue of DIO mice given CEF (50 and 500 mg/kg) and 10% OFS (data not shown). Serum amylase and lipase activities were elevated in some animals given VAN or CEF, but in the absence of microscopic changes in the pancreas, the significance is uncertain. No elevated signals were found among safety supported end points for the liver toxicity biomarkers and proteins aspartate aminotransferase (AST), alanine transaminase (ALT), total bilirubin, creatinine, creatine kinase, albumin, amylase, lipase, total protein and blood urea nitrogen (BUN) (data not shown).

Based on the observed changes in body weight and body fat, we decided to focus microbiome and biochemical analyses on the mice in the lean, vehicle, VAN 500 mg/kg (since significant changes in body weight or fat were not seen at the two lower VAN dosages), CEF 50 mg/kg, CEF 500 mg/kg and 10% OFS groups. Analysis of blood biochemistry suggests a specific effect of CEF is improved hyperglycemic control compared to 10% OFS or VAN dosed mice (n = 10 mice, except VAN 500 mg/kg where n = 9, with all comparisons to vehicle mice). Plasma glucose was significantly lower for both lean (P = 0.0388, Dunnett’s ANOVA) and CEF 500mg/kg (P = 0.0002) dosed mice compared to baseline vehicle mice (Fig 1E). Plasma triglycerides were significantly lower in CEF 500mg/kg (P = 0.0388, Dunnett’s ANOVA) and VAN 500 mg/kg (P = 0.0033) dosed mice (Fig 1F). Total cholesterol was also significantly lower in animal cohorts of lean (P < 0.0001, Dunnett’s ANOVA), 10% OFS (P = 0.0011) and CEF 50 mg/kg (P = 0.0271) but not in the higher CEF or VAN dosages (S1D Fig). Except for significantly lower HDL-cholesterol and HbA1 in lean mice (both P < 0.0001, Dunnett’s ANOVA) no significant changes were observed for other biochemical indices which included glycerol, plasma nonesterified fatty acids (NEFA), and glycated hemoglobin A1c (HbA1c) (data not shown).

**Fig 2:**
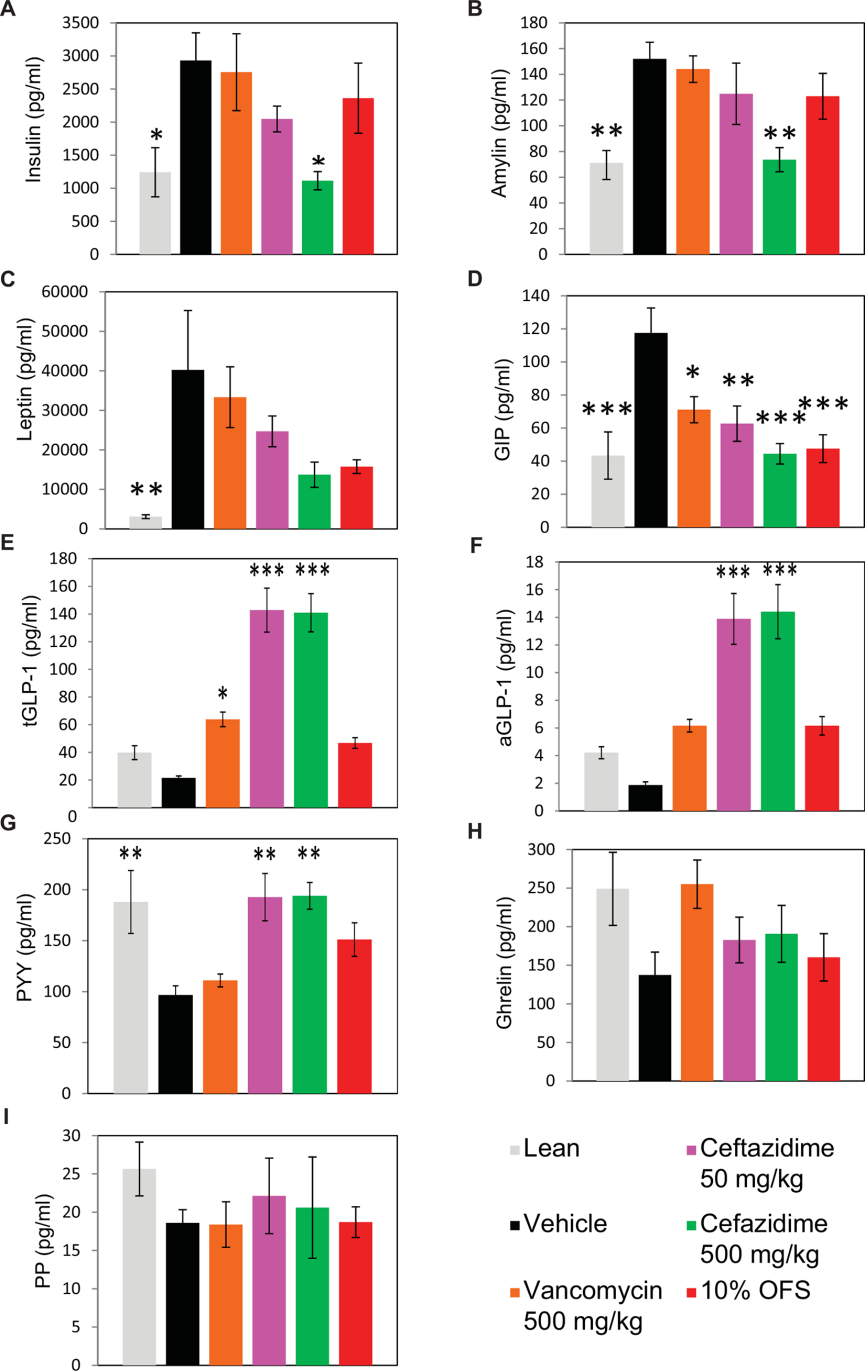
Changes in key metabolic peptides in mice at 14 days as measured for treatment cohorts lean diet, vehicle, vancomycin (500mg/kg), ceftazidime (50 mg/kg and 500 mg/kg) and 10% oligofructosaccharide (OFS). Measured peptides are (A) insulin, (B) amylin, (C) leptin, (D) gastric inhibitory polypeptide (GIP), (E) active glucagon-like peptide-1 (aGLP-1), (F) total glucagon-like peptide-1 (tGLP-1), (G) peptide tyrosine tyrosine (PYY), (H) ghrelin and, (I) pancreatic polypeptide (PP). * P < 0.05, ** P < 0.01, *** P < 0.001 obtained from ANOVA, Dunnett’s square. All error bars denote s.e.m.

Changes in key metabolic peptides were observed in relation to specific treatments, in particular CEF dosing. Hormone and peptide levels were analyzed at day 14 (end of study) for six treatment cohorts which were lean (n = 8), vehicle (n = 9), VAN 500 mg/kg (n = 9), CEF 50 mg/kg (n = 10), CEF 500 mg/kg (n = 8) and 10% OFS (n = 9; Fig 2A–2I). Insulin levels were significantly lower compared to vehicle in lean (P = 0.0265, Dunnett’s ANOVA) and CEF 500 mg/kg (P = 0.0150) treated animals (Fig 2A). Similarly, amylin was significantly lower in lean (P = 0.0041) and CEF 500 mg/kg (P = 0.0056) cohorts (Fig 2B). Lean mice had significantly lower leptin levels (P = 0.0051, Dunnett’s ANOVA; Fig 2C). Although leptin changes were not significantly lower for CEF 50 mg/kg, CEF 500 mg/kg or 10% OFS, the two antibiotic treatments trended lower. A significant dose-like response was seen for gastric inhibitory polypeptide (GIP) where significant lower levels were observed for lean P < 0.0001, Dunnett’s ANOVA), VAN 500 mg/kg (P = 0.0175), CEF 50 mg/kg (P = 0.0029), CEF 500 mg/kg (P < 0.0001) and 10% OFS (P < 0.0001) with the lowest and near equal levels for lean (mean 43.394 ± 11.51 pg/ml (s.e.m.)) and CEF 500 mg/kg (mean 44.464 ± 11.51 pg/ml (s.e.m.)) treatments (Fig 2D).

Interestingly, CEF 50mg/kg and CEF 500mg/kg dosed mice had significantly higher levels of glucagon-like peptide-1 (GLP-1) including total (tGLP-1) and active (aGLP-1) forms as well as protein tyrosine tyrosine (PYY). Relative to vehicle tGLP-1 (mean 21.517 ± 9.48 pg/ml (s.e.m.)) was elevated nearly seven-fold in CEF 50 mg/kg (mean 142.871 ± 9.0 pg/ml (s.e.m.); P < 0.0001; Dunnett’s ANOVA) and CEF 500 mg/kg (mean 140.989 ± 10.06 pg/ml (s.e.m.); P < 0.0001) treated animals while lean (mean 39.824 ± 10. 06 pg/ml (s.e.m); non-significant (NS)),VAN 500 mg/kg (mean 63.892 ± 9.483 pg/ml (s.e.m); P = 0.0121) and 10% OFS cohorts (mean 46.806 ± 9.48 pg/ml (s.e.m); NS) showed less change (Fig 2E). Similarly, aGLP-1had a greater than seven-fold significant increase from vehicle baseline (mean 1.877 ± 1.17 pg/ml (s.e.m.) in CEF 50 mg/kg (mean 13.89 ± 1.11 pg/ml (s.e.m.); P < 0.0001; Dunnett’s ANOVA) and CEF 500 mg/kg (mean 14.41 ± 1.24 pg/ml (s.e.m.); P < 0.0001) treated mice while aGLP-1 changes in lean, VAN 500 mg/kg and 10% OFS cohorts were non-significant (Fig 2F). PYY was significantly higher for lean (mean 188.003 ± 19.30 pg/ml (s.e.m.); P = 0.0055), CEF 50 mg/kg (mean 192.771 ±1 7.26 pg/ml (s.e.m.); P = 0.0017) and CEF 500 mg/kg (mean 194.036 ± 19.295 pg/ml (s.e.m.); P = 0.0028) cohorts compared to vehicle (mean 97.7 ± 18.191pg/ml (s.e.m.)) while VAN 500 mg/kg (mean 111.011 ± 18.191 pg/ml (s.e.m.); NS) and 10% OFS (mean 151.124 ± 18.191 pg/ml (s.e.m.); NS; Fig 2G) changes were not significant. Ghrelin (Fig 2H) and pancreatic polypeptide (PP; Fig 2I) also trended lower for CEF 50 mg/kg, CEF 500 mg/kg and 10% OFS relative to VAN 500 mg/kg and controls but not significantly.

### Ceftazidime effects in rat ZDF model

**Fig 3:**
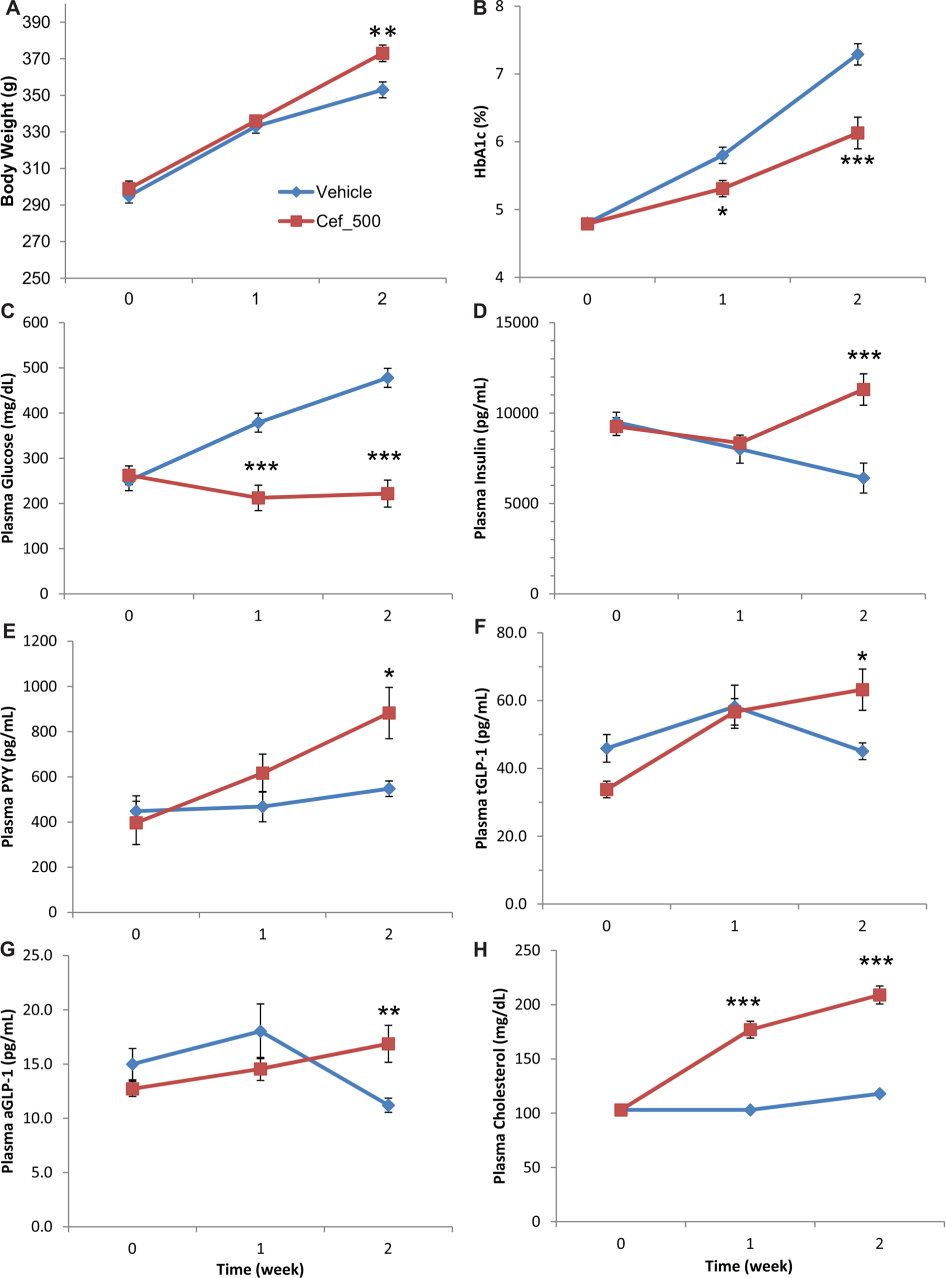
Changes in rat ZDF diabetes model after dosing with ceftazidime 500 mg/kg (Cef_500) over 2 weeks relative to vehicle treatment. Measured variables are; (A) body weight, (B) glycated hemoglobin (HbA1c) and, plasma (C) glucose, (D) insulin, (E) PYY, (F) tGLP-1, (G) aGLP-1 and, (H) cholesterol. * P < 0.05, ** P < 0.01, *** P < 0.001 obtained from ANOVA, Dunnett’s square. All error bars denote s.e.m.

To confirm our findings from the DIO mouse experiment, we tested the effect of CEF on hyperglycemia in the rat ZDF model which has a leptin gene deficiency (*lep-/lep-*). This 14 day study involved two groups of eight rats assigned as either vehicle controls or dosed with CEF 500mg/kg (see Materials and Methods). Biochemistry and phenotypic measures were taken at baseline, 7 days and end of study (for all time points n = 8; Fig 3A–3H and S2A–S2F Fig). Slightly higher and significant weight gain (P = 0.0041, Student’s t-test) was observed for CEF 500mg/kg dosed rats over controls (Fig 3A) that might be attributed to their improved glucose metabolism (see below). Similar changes have been observed with other anti-diabetic treatments (our unpublished data). Rats dosed at CEF 500mg/kg showed significant improvement in several glycemic variables. At the end of two weeks, CEF 500mg/kg dosed rats had significantly lower glycated hemoglobin (HbA1c; P = 0.0007, Student’s t-test) (Fig 3B) and plasma glucose levels (P < 0.0001) than the control group (Fig 3C). Significantly higher levels of insulin (P = 0.0078, Student’s t-test) (Fig 3D), PYY (P = 0.0107) (Fig 3E), tGLP-1 (P = 0.0007) (Fig 3F), aGLP-1(P = 0.0037) (Fig 3G) and cholesterol (P < 0.0001; Fig 3H) occurred in CEF 500 mg/kg treated rats by end of study. No significant changes between control and CEF 500 mg/kg treatments for GIP, NEFA, triglycerides and glycerol were observed (S2A–S2D Fig). However, CEF 500 mg/kg dosed rats did have significantly lower plasma (P = 0.003, Student’s t-test) and fecal bile acids (P < 0.001) relative to vehicle control (S2E and S2F Fig).

### Microbiome changes

At the end of study, mouse ceacum were removed from the six selected treatment groups for GIT microbiome metagenomic sequence analysis of the 16S rRNA variable regions V1 to V3 using the Roche 454 DNA sequencing platform and data analysis QIIME v1.8 software (see Materials and Methods) [22]. After removal of samples with read depths less than 500 DNA sequences and chimeric filtering, further statistical analysis were performed for lean (n = 9), vehicle (n = 9), 10% OFS (n = 9), VAN 500 mg/kg (n = 5), CEF 50 mg/kg (n = 5) and CEF 500 mg/kg (n = 7) treated mice.

**Fig 4:**
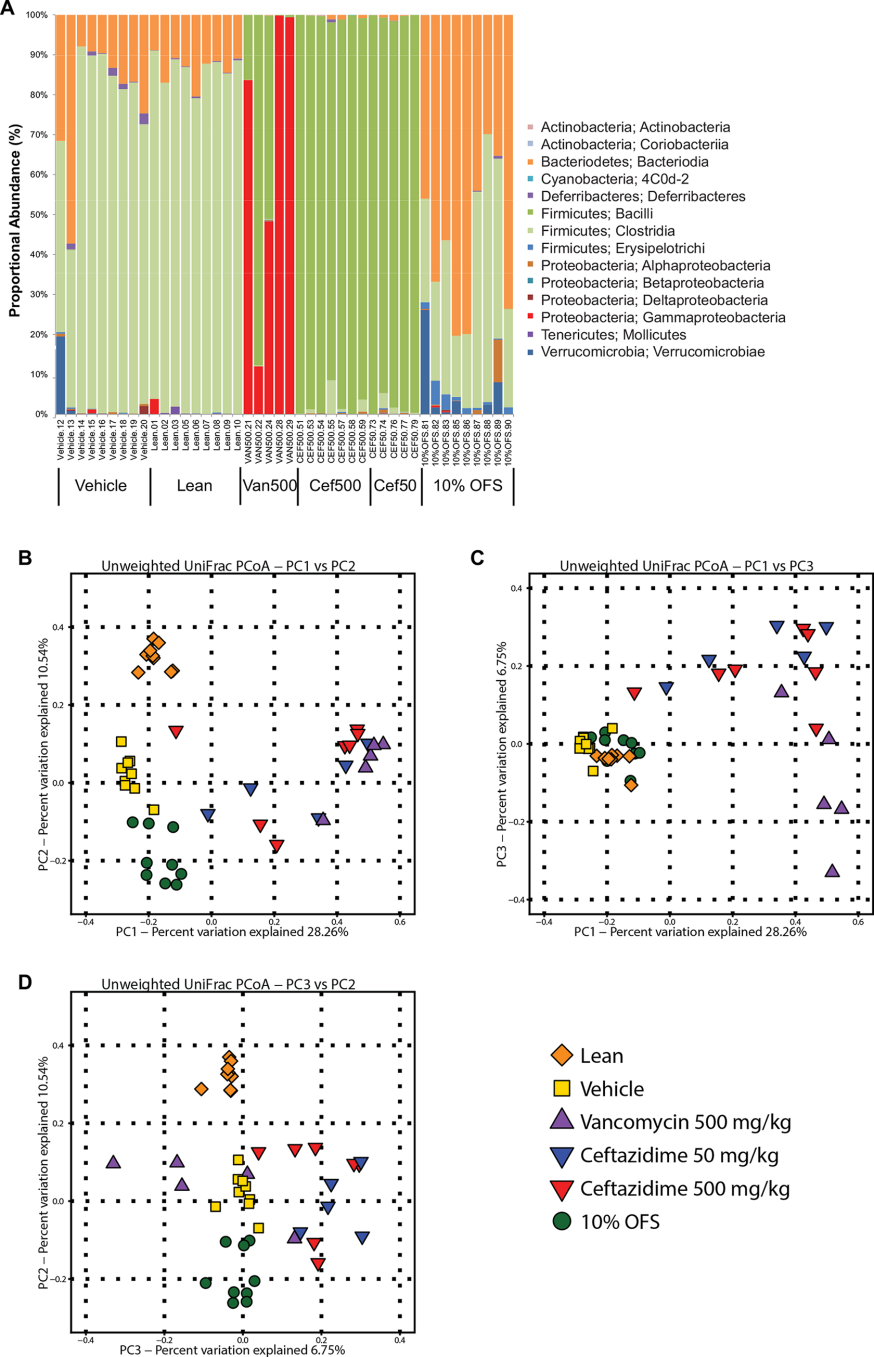
Mouse gut microbiome characterization based on 16S rRNA gene amplicon pyrosequencing. (A) Histogram of proportional changes in gut microbiota OTU abundance at the L3 level after 14 days as measured for treatment cohorts of lean, vehicle, vancomycin 500 mg/kg (VAN_500), ceftazidime 50 (CEF_50) and 500 mg/kg (CEF_500) and 10% OFS. DNA sequences are binned by species based on 16S RNA V1-V3 region with 97% identity cut-offs to Greengenes database (as implemented by QIIME software). Principal co-ordinate analysis (PCoA) of β-diversity measures of between-group microbiota community diversity based on treatment with plots of the principal components (PC); (B) PC1vs PC1, (C) PC3 vs. PC2 and, (D) PC1vs PC3.

Plots of alpha rarefraction curves (S3A Fig) and statistical analysis show that CEF treatments significantly lowered microbiome alpha diversity relative to the vehicle (P < 0.01, non-parametric t-test) while VAN and 10% OFS treatment (NS) did not. In lean and vehicle mouse microbiomes, Clostridia and Bacteroidia were the predominant microbiota classes while Bacteroidia trended higher than Clostridia in 10% OFS treated mice (Fig 4A; S1 Table). As expected, the antibiotic treated animals had microbial communities that reflected the anti-bacterial specificity of the drugs. VAN treated mice microbiomes were predominated by Gram-negative Proteobacteria. Low (50 mg/kg) and high (50 0mg/kg) CEF dosages resulted in microbiomes skewed towards Gram-positive Firmicutes distributions including Lactobacillales. There were no statistical significant differences in bacterial composition at the genus level between low and high CEF dosed mice (analysis not shown). The proportional distribution of classes of bacteria in mouse GIT microbiomes varied significantly (r^2^ = 0.38; P < 0.001; Adonis β diversity measure) according to treatment. Principal coordinate analysis (PCoA) plots of β diversity show distinct clusters for lean, vehicle,10% OFS and antibiotic cohorts with the latter showing some subtle differences between CEF versus VAN treatments (Fig 4B–4D).

Given that treatments of 10% OFS and CEF 500 mg/kg had comparable effects on weight loss yet different biochemical and hormonal profiles, we investigated the specific changes in the microbiome for these treatments relative to vehicle control. As expected from the known Gram-negative antibacterial spectrum of CEF, the microbiome of CEF 500 mg/kg treated mice had significantly (adjusted (adj.) P = 0.0097; ANOVA) increased proportional abundance of Lactobacillales and decreased abundances of Bacteroidetes and Clostridia (Table 1). Proteobacteria also trended lower just above the FDR adjusted P-value thresholds (unadjusted P = 0.01465; adj. P = 0.1086; ANOVA). In contrast, mice receiving 10% OFS had microbiomes characterized by much higher Bacteroidetes relative abundances and lower abundances of other Clostridiales groups (Table 2).

**Table 1:** Changes in microbiota proportional abundances in mice treated with ceftazidime (500 mg/kg) relative to vehicle control mice.

Operational Taxonomic Units (OTU) *	Mean Proportional Abundance		Percent	P-Value (C
	Ceftazidime (C) (n = 7)	Vehicle (V) (n = 9)	Change	vs. V) ^†^
Firmicutes; Clostridia; Clostridiales	0.0141	0.6123	-59.8267	0.0004
Firmicutes; Clostridia; Clostridiales; Ruminococcaceae; Oscillospira	0.0009	0.0668	-6.5915	0.0076
Firmicutes; Bacilli; Lactobacillales; Streptococcaceae; Lactococcus	0.6103	0.0000	61.0348	0.0097
Firmicutes; Clostridia; Clostridiales; Lachnospiraceae; [Ruminococcus]	0.0001	0.0089	-0.8830	0.0097
Bacteroidetes; Bacteroidia; Bacteroidales; S24-7	0.0009	0.0378	-3.6953	0.0097
Firmicutes; Bacilli; Bacillales; Bacillaceae	0.0044	0.0000	0.4405	0.0218
Firmicutes; Clostridia; Clostridiales; Ruminococcaceae; Ruminococcus	0.0006	0.0143	-1.3691	0.0218
Bacteroidetes; Bacteroidia; Bacteroidales; Rikenellaceae	0.0004	0.0526	-5.2168	0.0241

* OTUs determination at the L6 level in QIIME as described in the Methods.

† False Discovery Rate (FDR) corrected P-values for multiple ANOVA tests.

**Table 2:** Changes in microbiota proportional abundances in mice treated with 10% oligofructosaccharide (OFS) supplement relative to vehicle control mice.

Operational Taxonomic Units (OTU)*	Mean Proportional Abundance		Percent Change	P-Value (OFS vs. V) ^†^
	10% OFS (n = 9)	Vehicle (V) (n = 9)		
Firmicutes; Clostridia; Clostridiales	0.1248	0.6123	-48.7492	0.0076
Bacteroidetes; Bacteroidia; Bacteroidales; S24-7	0.3572	0.0378	31.9413	0.0076
Firmicutes; Clostridia; Clostridiales; Lachnospiraceae; [Ruminococcus]	0.0009	0.0089	-0.8056	0.0094
Bacteroidetes; Bacteroidia; Bacteroidales; Rikenellaceae	0.0058	0.0526	-4.6770	0.0404

* OTUs determination at the L6 level in QIIME as described in the Methods.

† False Discovery Rate (FDR) corrected P-values for multiple ANOVA tests.

We looked for potential functional changes in CEF dosed bacterial communities using the software PICRUSt [26] which uses 16S rRNA gene sequence profiles to estimate metagenomic content based on reference bacterial genomes and the KEGG pathway database [28]. The significantly increased pathways (adj. P < 0.05) were “phosphotransferase system (PTS)”, “phenylalanine, tyrosine and tryptophan biosynthesis”, “Ubiquinone and other terpenoid-quinone biosynthesis”, “Folate biosynthesis” and, “starch and sucrose metabolism” (S2 Table).

**Fig 5:**
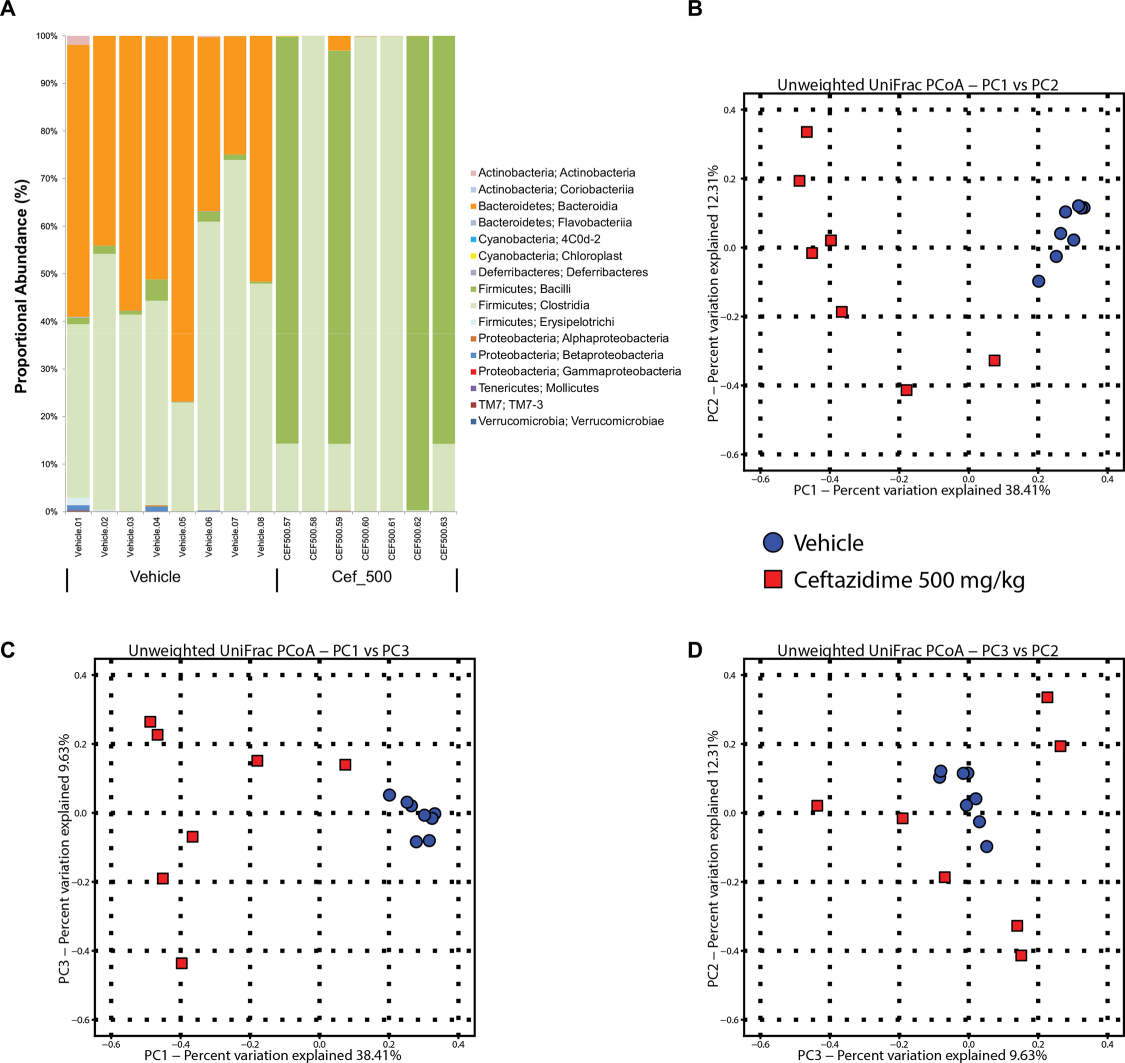
ZDF Rat microbiome characterization based on 16S rRNA gene amplicon pyrosequencing. (A) Histogram of proportional changes in gut microbiota OTU abundance at the L3 level after14 days as measured for treatment cohorts of vehicle (n = 8) and ceftazidime 500 mg/kg (n = 7). DNA sequences are binned by species based on 16S RNA V1-V3 region with 97% identity cut-offs to Greengenes database (as implemented by QIIME software [31]). Principal co-ordinate analysis (PCoA) of β-diversity measures of between-group microbiota community diversity based on treatment with plots of the principal components (PC); (B) PC1vs PC1, (C) PC3 vs. PC2 and, (D) PC1vs PC3.

Metagenomic analysis of rat feces using the same sequencing protocol (Roche 454 Flex DNA sequencing of 16S rRNA V1-V3 region) revealed comparable microbiomes between CEF 500 mg/kg treated DIO mice and ZDF rats (Fig 5A). Microbiome of rats receiving the vehicle were predominantly comprised of the phylum Bacteroidetes while the microbiome of CEF treated rats were mostly Firmicutes with a particularly significant increase (adj. P = 0.0295) in the relative abundance of the genus *Lactobacillus* (Table 3). Principal coordinate analysis (PCoA) plots of β diversity similarly showed distinct sample clusters for vehicle and antibiotic CEF 500 mg/kg treatment cohorts (Fig 5B–5D).

**Table 3:** Changes in microbiota proportional abundances in ZDF rats treated with ceftazidime (500 mg/kg) relative to vehicle control.

Operational Taxonomic Units (OTU) *	Mean Proportional Abundance		Percent Change	P-Value (C vs. V) ^†^
	Ceftazidime (C) (n = 7)	Vehicle (V) (n = 8)		
Bacteroidetes; Bacteroidia; Bacteroidales; Prevotellaceae; Prevotella	0.0038	0.4525	-44.8675	0.0002
Bacteroidetes; Bacteroidia; Bacteroidales; Bacteroidaceae; Bacteroides	0.0003	0.0152	-1.4893	0.0029
Firmicutes; Clostridia; Clostridiales; Ruminococcaceae; Oscillospira	0.0002	0.0104	-1.0181	0.0152
Bacteroidetes; Bacteroidia; Bacteroidales; S24-7;	0.0004	0.0233	-2.2853	0.0198
Bacteroidetes; Bacteroidia; Bacteroidales; Rikenellaceae;	0.0001	0.0066	-0.6515	0.0200
Firmicutes; Clostridia; Clostridiales; Clostridiaceae;	0.0000	0.0076	-0.7548	0.0218
Firmicutes; Clostridia; Clostridiales; Lachnospiraceae; Coprococcus	0.0000	0.1070	-10.6973	0.0257
Firmicutes; Clostridia; Clostridiales; Lachnospiraceae; Dorea	0.0000	0.0016	-0.1553	0.0261
Firmicutes; Clostridia; Clostridiales; Lachnospiraceae; Blautia	0.0001	0.0191	-1.9008	0.0285
Firmicutes; Bacilli; Lactobacillales; Lactobacillaceae; Lactobacillus	0.4971	0.0146	48.2593	0.0295
Firmicutes; Clostridia; Clostridiales; Lachnospiraceae;	0.0001	0.0481	-4.8006	0.0295
Firmicutes; Clostridia; Clostridiales; Lachnospiraceae; [Ruminococcus]	0.0000	0.0043	-0.4263	0.0300
Firmicutes; Clostridia; Clostridiales; Ruminococcaceae;	0.0001	0.1109	-11.0798	0.0307
Firmicutes; Clostridia; Clostridiales; Veillonellaceae; Phascolarctobacterium	0.0001	0.0059	-0.5725	0.0334

* OTUs determination at the L6 level in QIIME as described in the Methods.

† False Discovery Rate (FDR) corrected P-values for multiple ANOVA tests.

## Discussion

Microbiome composition has been associated with energy storage, fat turnover, intestinal integrity and inflammation which contribute to the regulation of weight and glycemic control. However, the causal relationship between microbiome composition and the severities of T2DM and/or obesity remains unclear with differing reports of bacterial species that are beneficial or detrimental to one or both conditions [32,11,10,33]. Antibiotics and prebiotics have been previously used to change microbiome composition in animal models of obesity and diabetes [34,15]. Limited human clinical observations suggest a link between antibiotics and metabolic diseases [35]. By measuring in a controlled diet-induced obesity (DIO) rodent model the biochemistry and phenotypic effects of two antibiotics with divergent anti-bacterial spectra as well as a prebiotic widely known to induce weight loss, our findings add new insights into the potential causal effects of microbiome community structures on the pathology of obesity and T2DM.

Our study suggests that highly distinct microbial species compositions are associated with combined accelerated weight loss and improved hyperglycemic control versus weight loss alone. Relative to controls, mice treated with 10% OFS showed greater weight loss and an elevated relative abundance of Bacteroidetes species but no significant changes in glucose, insulin or anti-diabetic hormones such as GLP-1 and PYY. However, the antibiotic CEF with its mainly anti-Gram-negative bacteria spectrum caused both weight loss as well as a favorable glycemic profile of glucose, insulin, aGLP-1, tGLP-1 and PYY levels. Conversely, the Gram-positive spectrum antibiotic VAN did not significantly change weight loss or improve hyperglycemic control.

Energy homeostasis is regulated by a number of factors such as neurotransmitters, hormones and others through central and peripheral mechanisms. The neuropeptide Y (NPY) system is regarded as one of the main systems that play a significant role in energy homeostasis. For example, PYY, which is expressed mainly by the enteroendocrine cells, plays a significant role in mediating satiety, while several other members of the NPY system promote feeding [36]. PYY releasing enteroendocrine cells are influenced directly by metabolic intermediates such as short chain fatty acids, the generation of which could also be influenced by gut microbiota from various forms of dietary fibers [37]. Post prandial secretion of incretin hormones such as GLP-1 has been considered as an important regulator of satiety through melanocortin 4 receptor mediated neuronal activity [38]. Increases in GLP-1 levels either through inhibitors of dipeptideylpeptidase 4 or long acting GLP-1 receptor agonists contributes to the reduction of plasma triglyceride levels as well as stores of triglycerides in the hepatic system. GLP-1 is secreted by the L-cells of the small intestine, and is attributed to play a role in lipid absorption as well. It is interesting that both of these important hormones (GLP-1 and PYY) increased in animals treated with CEF and may have contributed to the observed metabolic improvements.

Given that both antibiotics would lower overall intestinal bacterial abundance and diversity, the mechanistic effects of CEF cannot be attributed to general “cleansing” of the GIT microbiome. Instead, CEF effects of steady weight loss and improved hyperglycemic control appear to be broadly related to changes in the gut microbiome composition. CEF treated mice had higher proportions of Gram-positive bacteria, the Firmicutes, and lower abundances of Gram-negative bacteria especially the Proteobacteria. Comparing the two dosing CEF regimens, the highest CEF (500mg/kg) dosing caused greater changes in body weight and fat compared to the lowest CEF (50mg/kg) dosing. The microbiome composition at the genus level (L6) between those two dosing regimens was not significantly different (based on P- values after FDR correction–analysis not shown). There were subtle abundance differences in several genera which might warrant further investigation since low abundance species could still have significant metabolic impacts (S1 Table). On the other hand, the similarities of aGLP-1, tGLP1 and PYY levels across both CEF dosing regimens seem to correspond with the observed common microbiota community structures.

Previous studies in mice show that high-fat diet increases the proportion of Gram-negative to Gram-positive microbes leading to elevated bacterial lipopolysaccharides (LPS) and pro-inflammatory responses [39]. In our study, 10% OFS treated mice had a higher relative abundance of Bacteroidetes which might account for the weight loss effects of this prebiotic. Higher Bacteroidetes levels have been reported for microbiomes of lean human individuals compared to their obese twin counterpart (and this result was recapitulated in germ-free mice after transplantation of human lean twin fecal samples) [5]. Other prebiotics such as fructans appear to boost Gram-positive bacteria levels and increase GLP-1 and PYY in healthy human subjects [40,41]. Further studies into the effects of different prebiotic supplements on the microbiome are needed.

The impact of antibiotics on metabolic profiles in animals and humans also requires further investigation. Murphy and colleagues reported some weight loss and improvement in fasting blood glucose levels but not insulin levels in DIO mouse models after administration of VAN [42]. Conversely, VAN treatment of patients with infective endocarditis has been linked to newly acquired obesity [35]. Membrez et al. found that norfloxacin and ampicillin enhanced glucose tolerance in obese and insulin-resistant *ob-/ob-* mice [34]. In our study, we also saw a slight weight drop in VAN dosed animals relative to the vehicle control although CEF caused more pronounced changes. To our knowledge, the effect of CEF or any cephalosporin on the microbiome in conjunction with metabolic diseases has not been previously reported. We suggest that CEF might be a valuable tool compound for the *in vivo* modulation of the GIT microbiome in future animal studies.

Our study provides further insight into the specific changes in the host which might be modulated by the gut microbiome. In both the mouse DIO and rat ZDF models, CEF treatment lead to a significant induction of the peptides tGLP-1, aGLP-1 and PYY. Both animal models when treated with CEF displayed decreased glucose levels relative to the controls although concurrent weight loss was observed in the DIO mouse but not the ZDF rat experiments, where there was a slight but significant weight gain. There could be several reasons for the opposing directionality in weight gain/loss such as differences in the models in terms of dietary (DIO mice) versus genetic modulation (ZDF rats have a leptin gene deficiency). However, the two independent animal experiments do provide support to the hypothesis that the glucose effect could be body weight/food intake-independent.

Current theory suggests that modulation of inflammatory responses by gut microbiota might be the central axis of many chronic diseases, particularly obesity and diabetes [43]. Specific bacterial metabolites and secreted peptides interacting with human receptors such as GPCR43 potentially either activate or suppress inflammatory responses [44]. Gut bacterial LPS, a known pro-inflammatory factor, is produced by Gram-negative bacteria such as *Escherichia coli* and other Proteobacteria [45]. The Gram-negative anti-bacterial spectrum of CEF would lead to the suppression of LPS-producers which might partially account for the drug’s positive effects on obesity and diabetic factors. Bile acids and short chain fatty acids (SCFAs) produced by bacterial metabolism are also implicated as immunomodulators [46]. We measured changes in bile acids in the rat ZDF experiment (but not DIO mice) and found a significant drop following CEF treatment, suggesting potential activation of host-microbe cross-talk pathways.

Our study suggests that targeted manipulation of the gut microbiota using narrow-spectrum antibiotics can reveal the specific changes in the microbiome associated with disease pathology. We suggest that such chemogenomic approaches to selectively disrupting mammalian microbial communities *in vivo* can illuminate crucial cause-effect linkages between the microbiome and human health. While long term clinical use of antibiotics as treatment paradigm for obesity and diabetes diseases has several practical drawbacks, such as possible toxic side-effects and the propagation of antibiotic resistant strains [47], we demonstrate that modulating the gut microbial ecosystem is a potential approach for understanding and treating chronic metabolic diseases.

## Supporting Information

S1 Fig Additional data for mouse cohorts on treatments of lean diet, vehicle, vancomycin (500mg/kg), ceftazidime (50 mg/kg and 500 mg/kg) and 10% oligofructosaccharide (OFS). Plots show; (A) food intake measured at intervals over the 14 day study, (B) total food intake, (C) percentage of body fat at baseline and end of study (Day 14) and (D) cholesterol at end of study. Denoted by “*” are significant changes versus vehicle (P ≤ 0.05 ANOVA, Dunnett’s square) with n = 8 for each treatment.
(EPS)

S2 Fig Additional data about changes in rat ZDF diabetes model after dosing with ceftazidime 500 mg/kg (CEF_500) over 2 weeks relative to vehicle treatment. Measured variables are plasma; (A) GIP, (B) triglycerides, (C) nonesterified fatty acids (NEFA) and, (D) glycerol. Also shown are changes in; (E) total fecal bile acids (BA) and, (F) plasma BA. Significant changes versus vehicle are denoted for as * = P ≤ 0.05; ** = P ≤ 0.01 and *** = P ≤ 0.001 (ANOVA, Dunnett’s square) with n = 8 for each treatment.
(EPS)

S3 Fig Alpha diversity rarefaction curves for 16S rRNA sequences. Rarefaction curves based on chao1 measure for (A) mouse and, (B) rat microbiome analyses.
(EPS)

S1 Table Mean proportional occurrences of bacteria at L6 level (genus, using closed reference calling in QIIME v1.9 [31]) across different treatments and dosages. (XLSX)

S2 Table Bacterial biochemical pathways suggested to be significantly up-regulated in ceftazidime treated mice according to PICRUSt software [26]. (XLSX)

## References

[1] WHO. World Health Organization: obesity and overweight—fact sheet No.311. 2012;

[2] Holmes E, Kinross J, Gibson GR, Burcelin R, Jia W, Pettersson S et al Therapeutic modulation of microbiota-host metabolic interactions. Sci Transl Med. 2012; 4:137rv6 10.1126/scitranslmed.3004244 22674556

[3] Delzenne NM, Neyrinck AM, Backhed F, Cani PD. Targeting gut microbiota in obesity: effects of prebiotics and probiotics. Nat Rev Endocrinol. 2011; 7:639–46. 10.1038/nrendo.2011.126 21826100

[4] Turnbaugh PJ, Ley RE, Mahowald MA, Magrini V, Mardis ER, Gordon JI. An obesity-associated gut microbiome with increased capacity for energy harvest. Nature. 2006; 444:1027–31. 17183312 10.1038/nature05414

[5] Ridaura VK, Faith JJ, Rey FE, Cheng J, Duncan AE, Kau AL et al Gut microbiota from twins discordant for obesity modulate metabolism in mice. Science. 2013; 341:1241214 10.1126/science.1241214 24009397 PMC3829625

[6] Le CE, Nielsen T, Qin J, Prifti E, Hildebrand F, Falony G et al Richness of human gut microbiome correlates with metabolic markers. Nature. 2013; 500:541–6. 10.1038/nature12506 23985870

[7] Karlsson FH, Tremaroli V, Nookaew I, Bergstrom G, Behre CJ, Fagerberg B et al Gut metagenome in European women with normal, impaired and diabetic glucose control. Nature. 2013; 498:99–103. 10.1038/nature12198 23719380

[8] Qin J, Li Y, Cai Z, Li S, Zhu J, Zhang F et al A metagenome-wide association study of gut microbiota in type 2 diabetes. Nature. 2012; 490:55–60. 10.1038/nature11450 23023125

[9] Sweeney TE, Morton JM. The human gut microbiome: a review of the effect of obesity and surgically induced weight loss. JAMA Surg. 2013; 148:563–9. 10.1001/jamasurg.2013.5 23571517 PMC4392891

[10] Duncan SH, Lobley GE, Holtrop G, Ince J, Johnstone AM, Louis P et al Human colonic microbiota associated with diet, obesity and weight loss. Int J Obes (Lond). 2008; 32:1720–4. 18779823 10.1038/ijo.2008.155

[11] Turnbaugh PJ, Hamady M, Yatsunenko T, Cantarel BL, Duncan A, Ley RE et al A core gut microbiome in obese and lean twins. Nature. 2009; 457:480–4. 10.1038/nature07540 19043404 PMC2677729

[12] Derrien M, van BP, Hooiveld G, Norin E, Muller M, de Vos WM. Modulation of mucosal immune response, tolerance, and proliferation in mice colonized by the mucin-degrader *Akkermansia muciniphila* . Front Microbiol. 2011; 2:166 10.3389/fmicb.2011.00166 21904534 PMC3153965

[13] van Passel MW, Kant R, Zoetendal EG, Plugge CM, Derrien M, Malfatti SA et al The genome of *Akkermansia muciniphila*, a dedicated intestinal mucin degrader, and its use in exploring intestinal metagenomes. PLoS One. 2011; 6:e16876 10.1371/journal.pone.0016876 21390229 PMC3048395

[14] Everard A, Belzer C, Geurts L, Ouwerkerk JP, Druart C, Bindels LB et al Cross-talk between *Akkermansia muciniphila* and intestinal epithelium controls diet-induced obesity. Proc Natl Acad Sci U S A. 2013; 110:9066–71. 10.1073/pnas.1219451110 23671105 PMC3670398

[15] Membrez M, Blancher F, Jaquet M, Bibiloni R, Cani PD, Burcelin RG et al Gut microbiota modulation with norfloxacin and ampicillin enhances glucose tolerance in mice. FASEB J. 2008; 22:2416–26. 10.1096/fj.07-102723 18326786

[16] Delzenne NM, Cani PD, Neyrinck AM. Modulation of glucagon-like peptide 1 and energy metabolism by inulin and oligofructose: experimental data. J Nutr. 2007; 137:2547S–51S. 17951500 10.1093/jn/137.11.2547S

[17] Meyer D, Stasse-Wolthuis M. The bifidogenic effect of inulin and oligofructose and its consequences for gut health. Eur J Clin Nutr. 2009; 63:1277–89. 10.1038/ejcn.2009.64 19690573

[18] Moellering RC Jr. Vancomycin: a 50-year reassessment. Clin Infect Dis. 2006; 42 Suppl 1:S3–S4. 16323117 10.1086/491708

[19] Hayes MV, Orr DC. Mode of action of ceftazidime: affinity for the penicillin-binding proteins of *Escherichia coli* K12, *Pseudomonas aeruginosa* and *Staphylococcus aureus* . J Antimicrob Chemother. 1983; 12:119–26. 6413485 10.1093/jac/12.2.119

[20] Wang B, Chandrasekera PC, Pippin JJ. Leptin- and leptin receptor-deficient rodent models: relevance for human type 2 diabetes. Curr Diabetes Rev. 2014; 10:131–45. 24809394 10.2174/1573399810666140508121012 PMC4082168

[21] Napolitano A, Miller S, Nicholls AW, Baker D, Van HS, Thomas E et al Novel gut-based pharmacology of metformin in patients with type 2 diabetes mellitus. PLoS One. 2014; 9:e100778 10.1371/journal.pone.0100778 24988476 PMC4079657

[22] Navas-Molina JA, Peralta-Sanchez JM, Gonzalez A, McMurdie PJ, Vazquez-Baeza Y, Xu Z et al Advancing our understanding of the human microbiome using QIIME. Methods Enzymol. 2013; 531:371–444. 10.1016/B978-0-12-407863-5.00019-8 24060131 PMC4517945

[23] Haas BJ, Gevers D, Earl AM, Feldgarden M, Ward DV, Giannoukos G et al Chimeric 16S rRNA sequence formation and detection in Sanger and 454-pyrosequenced PCR amplicons. Genome Res. 2011; 21:494–504. 10.1101/gr.112730.110 21212162 PMC3044863

[24] Edgar RC. Search and clustering orders of magnitude faster than BLAST. Bioinformatics. 2010; 26:2460–1. 10.1093/bioinformatics/btq461 20709691

[25] DeSantis TZ, Hugenholtz P, Larsen N, Rojas M, Brodie EL, Keller K et al Greengenes, a chimera-checked 16S rRNA gene database and workbench compatible with ARB. Appl Environ Microbiol. 2006; 72:5069–72. 16820507 10.1128/AEM.03006-05 PMC1489311

[26] Langille MG, Zaneveld J, Caporaso JG, McDonald D, Knights D, Reyes JA et al Predictive functional profiling of microbial communities using 16S rRNA marker gene sequences. Nat Biotechnol. 2013; 31:814–21. 10.1038/nbt.2676 23975157 PMC3819121

[27] Kanehisa M, Goto S, Sato Y, Kawashima M, Furumichi M, Tanabe M. Data, information, knowledge and principle: back to metabolism in KEGG. Nucleic Acids Res. 2014; 42:D199–D205. 10.1093/nar/gkt1076 24214961 PMC3965122

[28] Kanehisa M, Goto S. KEGG: kyoto encyclopedia of genes and genomes. Nucleic Acids Res. 2000; 28:27–30. 10592173 10.1093/nar/28.1.27 PMC102409

[29] Benjamini Y, Hochberg Y. Controlling the false discovery rate: a practical and powerful approach for multiple testing. Journal of the Royal Statistical Society, Series B. 1995; 57:289–300.

[30] R Core Team. R: A language and environment for statistical computing. 2013;

[31] Caporaso JG, Kuczynski J, Stombaugh J, Bittinger K, Bushman FD, Costello EK et al QIIME allows analysis of high-throughput community sequencing data. Nat Methods. 2010; 7:335–6. 10.1038/nmeth.f.303 20383131 PMC3156573

[32] Cotillard A, Kennedy SP, Kong LC, Prifti E, Pons N, Le CE et al Dietary intervention impact on gut microbial gene richness. Nature. 2013; 500:585–8. 10.1038/nature12480 23985875

[33] Ley RE, Turnbaugh PJ, Klein S, Gordon JI. Microbial ecology: human gut microbes associated with obesity. Nature. 2006; 444:1022–3. 17183309 10.1038/4441022a

[34] Chou CJ, Membrez M, Blancher F. Gut decontamination with norfloxacin and ampicillin enhances insulin sensitivity in mice. Nestle Nutr Workshop Ser Pediatr Program. 2008; 62:127–37. 10.1159/000146256 18626197

[35] Thuny F, Richet H, Casalta JP, Angelakis E, Habib G, Raoult D. Vancomycin treatment of infective endocarditis is linked with recently acquired obesity. PLoS One. 2010; 5:e9074 10.1371/journal.pone.0009074 20161775 PMC2818846

[36] Loh K, Herzog H, Shi YC. Regulation of energy homeostasis by the NPY system. Trends Endocrinol Metab. 2015; 26:125–35. 10.1016/j.tem.2015.01.003 25662369

[37] Holzer P, Farzi A. Neuropeptides and the microbiota-gut-brain axis. Adv Exp Med Biol. 2014; 817:195–219. 10.1007/978-1-4939-0897-4_9 24997035 PMC4359909

[38] Dallinga-Thie GM, Nieuwdorp M. GLP1, an important regulator of intestinal lipid metabolism. Arterioscler Thromb Vasc Biol. 2015; 35:1048–9. 10.1161/ATVBAHA.115.305479 25903650

[39] Cani PD, Neyrinck AM, Fava F, Knauf C, Burcelin RG, Tuohy KM et al Selective increases of bifidobacteria in gut microflora improve high-fat-diet-induced diabetes in mice through a mechanism associated with endotoxaemia. Diabetologia. 2007; 50:2374–83. 17823788 10.1007/s00125-007-0791-0

[40] Cani PD, Lecourt E, Dewulf EM, Sohet FM, Pachikian BD, Naslain D et al Gut microbiota fermentation of prebiotics increases satietogenic and incretin gut peptide production with consequences for appetite sensation and glucose response after a meal. Am J Clin Nutr. 2009; 90:1236–43. 10.3945/ajcn.2009.28095 19776140

[41] Tarini J, Wolever TM. The fermentable fibre inulin increases postprandial serum short-chain fatty acids and reduces free-fatty acids and ghrelin in healthy subjects. Appl Physiol Nutr Metab. 2010; 35:9–16. 10.1139/H09-119 20130660

[42] Murphy EF, Cotter PD, Hogan A, O'Sullivan O, Joyce A, Fouhy F et al Divergent metabolic outcomes arising from targeted manipulation of the gut microbiota in diet-induced obesity. Gut. 2013; 62:220–6. 10.1136/gutjnl-2011-300705 22345653

[43] Cox AJ, West NP, Cripps AW. Obesity, inflammation, and the gut microbiota. Lancet Diabetes Endocrinol. 2014; 10.1016/S2213-8587(14)70134-2 25066177

[44] Kimura I, Ozawa K, Inoue D, Imamura T, Kimura K, Maeda T et al The gut microbiota suppresses insulin-mediated fat accumulation via the short-chain fatty acid receptor GPR43. Nat Commun. 2013; 4:1829 10.1038/ncomms2852 23652017 PMC3674247

[45] Ueda Y, Kayama H, Jeon SG, Kusu T, Isaka Y, Rakugi H et al Commensal microbiota induce LPS hyporesponsiveness in colonic macrophages via the production of IL-10. Int Immunol. 2010; 22:953–62. 10.1093/intimm/dxq449 21051439

[46] Smith PM, Howitt MR, Panikov N, Michaud M, Gallini CA, Bohlooly Y et al The microbial metabolites, short-chain fatty acids, regulate colonic Treg cell homeostasis. Science. 2013; 341:569–73. 10.1126/science.1241165 23828891 PMC3807819

[47] Blaser M. Antibiotic overuse: Stop the killing of beneficial bacteria. Nature. 2011; 476:393–4. 10.1038/476393a 21866137

